# Partial Achilles Tendon Rupture—A Neglected Entity: A Narrative Literature Review on Diagnostics and Treatment Options

**DOI:** 10.3390/jcm9103380

**Published:** 2020-10-21

**Authors:** Matthias Gatz, Christoph Spang, Håkan Alfredson

**Affiliations:** 1 Department of Orthopedics, University Hospital RWTH Aachen, 52074 Aachen, Germany; 2 Department of Integrative Medical Biology, Anatomy Section, Umeå University, 90187 Umeå, Sweden; christoph.spang@umu.se; 3 Alfen Spine Center, 97080 Würzburg, Germany; 4 Sports Medicine Unit, Department of Community Research and Rehabilitation, Umeå University, 90187 Umeå, Sweden; hakan.alfredson@umu.se; 5 Institute of Sports Exercise and Health, University College London Hospitals, London W1T 7HA, UK

**Keywords:** partial rupture, partial tear, achilles, tendinopathy

## Abstract

Partial ruptures in the Achilles tendon are rather uncommon and are often misinterpreted as aggravated Achilles tendinopathy, and not always considered as a differential diagnosis. The aim of this literature review was to characterize typical symptoms, to provide an overview of available diagnosis and treatment options, and to give reference points for future research. There were few studies and sparse knowledge of scientific value, making it difficult to give evidence-based recommendations. Based on the few studies and the authors’ clinical experience, a diagnosis should be based on a patient’s history with a typical sharp onset of pain and inability to fully load the tendon. Previous intratendinous cortisone injections might be present. Clinical findings are a localized tender region in the tendon and often weakness during heel raises. Ultrasound and Doppler examinations show a region with an irregular and bulging superficial tendon line, often together with localized high blood flow. Magnetic resonance Imaging (MRI) shows a hyperintense signal in the tendon on T1 and T2-weighted sequences. First-line therapy should be a conservative approach using a 2 cm heel lift for the first 6 weeks and avoiding tendon stretching (for 12 weeks). This is followed by a reduced heel lift of 1 cm and progressive tendon loading at weeks 7–12. After 12 weeks, the heel lift can be removed if pain-free, and the patient can gradually start eccentric exercises lowering the heel below floor level and gradually returning to previous sport level. If conservative management has a poor effect, surgical exploration and the excision of the partial rupture and suturing is required. Augmentation procedures or anchor applications might be useful for partial ruptures in the Achilles insertion, but this depends on the size and exact location. After surgery, the 12 to 14-week rehabilitation program used in conservative management can be recommended before the patient’s return to full tendon loading activities.

## 1. Introduction

Achilles tendinopathy is the most common reason for Achilles tendon pain and can be categorized into insertional, midportion, or plantaris tendon-related Achilles tendinopathy [1]. A further pathology is the acute Achilles tendon rupture with a sudden onset of pain and functional disability. For both Achilles tendinopathy and Achilles tendon rupture, there are several evidence-based nonsurgical and surgical treatment options and diagnostic pathways based on meta-analyses, randomized controlled trials, and consensus statements [2,3,4].

In comparison, a partial Achilles tendon tear is rather uncommon, is not always considered as a differential diagnosis, and might be misinterpreted as aggravated Achilles tendinopathy [5]. While Achilles tendinopathy and Achilles tendon rupture can be easily differentiated from each other, partial Achilles tendon rupture meets the symptom criteria of both [6,7]. This might lead to difficulties in diagnosing and choosing the most appropriate treatment option (e.g., load vs. immobilization). The physician might end up being caught between two stools. To the best of our knowledge, there is no consensus regarding diagnosis and treatment for partial Achilles ruptures.

The aim of this literature review is to characterize typical symptoms, to provide an overview of available diagnosis and treatment options, and to give reference points for future research.

## 2. Methods

The literature search included acute partial Achilles tendon rupture as well as partial tendon tears together with a history of tendinopathy. A review of the literature was performed including all articles published in PubMed, Scopus, and Google Scholar until August 2020. The following search terms were used alone and in combination: “Achilles”, “partial”, “partial rupture”, and “partial tear”; and the articles were evaluated with regard to the following research aspects: definition, epidemiology, etiology, pathogenesis, diagnosis, and treatment.

Articles of all clinical trial levels in English, German, Swedish, and Portuguese were included. After assessing all abstracts (*n* = 388), the full text and the bibliographies of the relevant articles were analyzed with regard to the questions posed (*n* = 56). Articles including animal or biomechanical studies or outcomes without the area of interest were excluded (*n* = 22). Due to the advancements promoted by the scientific progress of imaging modalities, surgical techniques, and improved understanding of tendon load for rehabilitation, mainly articles from the years 1990–2020 were included. However, after scanning bibliographies of the relevant articles (*n* = 4), articles published before 1990 were added. The final literature research revealed 38 relevant articles.

## 3. Results

### 3.1. Definitions

A partial tear is defined as a partial discontinuation of the Achilles tendon and usually has an acute onset. There is often pain during loading and a feeling of weakness. Patients typically maintain the ability to train but do not reach maximal loading [8]. Physically active patients suffer from permanent symptoms (39%) or initial warm-up pain after a period of resting (61%), which might decrease or increase during physical activity [6]. Tendon thickening, localized pain at a specific region, occasionally a palpable tendon discontinuity, loss of function, and limping are clinical findings [6,9,10].

### 3.2. Etiology

Most partial tears are caused by an overload of the tendon tissue and occur in areas afflicted with tendinopathic tissue changes [11]. The precise relationship between tendinopathy and partial tears and the pathogenesis are still not fully understood. According to recent studies, the pathogenesis of tendinopathy is more likely to be a result of altered tissue homeostasis by repeated mechanical overload, rather than a repair response to a partial rupture in the tendon [12]. Thus, it seems more likely that partial ruptures appear in tissue areas that had previously developed tendinopathic features. In fact, in recent studies non-homogenous stress at subclinical or clinically symptomatic tendinopathic tissue is seen as the underlying mechanism for the development of an acute partial rupture, mainly in the midportion of the Achilles tendon [13]. At the tendon insertion, partial ruptures might additionally be caused by a tendon impingement of bony prominences in the calcaneus [14].

Injections into the tendon, especially cortisone, are known to have a significant influence on the development of partial and total tendon ruptures by starting local degenerative processes [15]. In some studies, a previous intratendinous cortisone injection was observed in nearly 50% of partial ruptures [9,10]. Similar figures were seen for partial tears and the use of fluorquinolone medication [16].

In general, for surgical reconstruction it has to be considered that the Achilles tendon is formed by the confluence of the tendons of the soleus and both gastrocnemius muscles. These subtendons intertwine and twist towards their distal insertion and have a specific arrangement in the midportion and the insertion of the calcaneus [5,17]. Based on the anatomic region within the tendon, partial ruptures might be assigned to the specific subtendon, which might lead to an isolated partial hypotrophy of the gastrocsoleus muscle complex with reduced voluntary electromyography activity [17]. Partial tears mostly occur in the posterior mid-tendon 3–4 cm above the superior calcaneus, whereas a further study demonstrated that intratendinous tears are mostly anterior and medial, which mostly present fibers of the soleus and lateral gastrocnemius components [8,18,19].

### 3.3. Classification

Smigielski classified partial injuries of the Achilles tendon based on histopathological aspects with further subgroups based on the amount of damaged bundles and the exact origin of the gastrocsoleus complex [5]:

(a) acute injury with fresh collagen disruption representing an acute microinjury, with detectable extravascular erythrocytes and an early reparative process.
(b) chronic injury with fatty metaplasia and infiltrated vessels showing a reduced potential for self-healing.

Recently, it has been suggested to distinguish between intratendinous tears and partial tears by defining the latter as extending to the periphery of the tendon, in contrast to an intratendinous tear, which does not reach the peripheral edge of the tendon and extends in the majority of cases longitudinally [8].

In histopathological analysis, degenerative changes, such as collagen disorientation and fiber separation, with increased mucoid ground substance, fibrin deposits, hypercellularity, and neovascularization are present in partial ruptures [11]. Since these are also current findings in tendinosis, it has been advocated that partial ruptures are rather microlesions representing an advanced stage of tendinosis [11]. However, granulation tissue, fibroblastic and myofibroblastic proliferation, and hemorrhage are more frequently associated with partial ruptures than with tendinopathy [11].

### 3.4. Epidemiology

Affected patients are mostly younger males with a higher sports level than patients with midportion tendinopathy or Achilles tendon rupture [8,9,11,20,21,22]. However, in top athletes, partial ruptures might not be that common. A Champions League injury study with an 11-year follow-up stated a total of seven total ruptures and two partial ruptures. Furthermore, at the London 2012 Olympics, four partial ruptures and one total rupture were seen in an imaging study [23].

In some imaging studies, partial rupture might be present in up to 25% of cases with Achilles tendinopathy, but a possible selection bias has to be considered since a further imaging study reported an incidence rate of only 1.6% [24]. Additionally, studies on surgically treated patients with Achilles tendinopathy have reported a high partial rupture rate of 19% and 25%, but also a lower rate of 4% in a large cohort with 771 surgically treated tendons [1,11,22].

**Figure 1:**
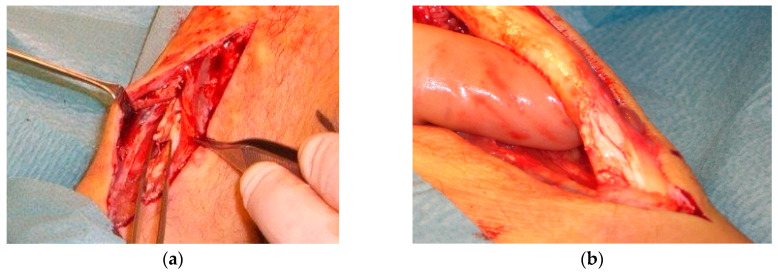
(**a**) Surgical observations in patients with partial Achilles tendon ruptures; (**b**) higher magnification of longitudinal partial Achilles tendon rupture.

Therefore, the exact prevalence is unknown and might differ between several cohorts. Furthermore, the discrepancy between the studies might be due to different definitions of a partial rupture and the method of detection. In surgical studies, the partial rupture is often diagnosed by the surgeon based on how the tissue looks macroscopically (Figure 1). Some studies use ultrasound or MRI for diagnosis.

### 3.5. Imaging

Differentiation between full-thickness tears and partial tears can be adequately assessed with ultrasound (*n* = 26, accuracy 92%, sensitivity 100%, and specificity 83%), showing a significant difference in tendon thickness, an increased posterior acoustic shadowing at the side of tendon abnormality, and tendon retraction in case of a full-thickness tear [7]. Comparing findings of preoperative ultrasound to findings at surgery, Kälebo et al. reported an excellent diagnostic accuracy of 95% for ultrasound (sensitivity 0.94; specificity 1.00) [25]. Further studies revealed corresponding preoperative findings in 8/11 patients (sensitivity 72%) or 10/11 (sensitivity 90%) [26,27]. However, these good results might be based on a selection bias in a surgically treated, small collective, which might have larger and hence easier-to-detect partial ruptures.

**Figure 2:**
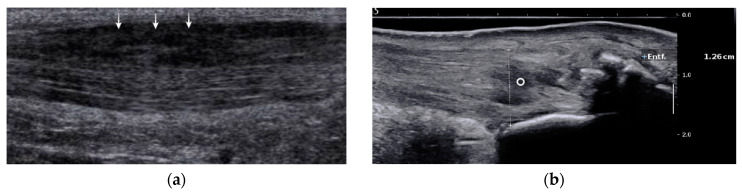
US images of partial Achilles tendon ruptures: (**a**) partial rupture in the dorsal part of the Achilles tendon midportion (arrows); (**b**) partial rupture in insertional area of the Achilles tendon (circle). US; ultrasound.

**Figure 3:**
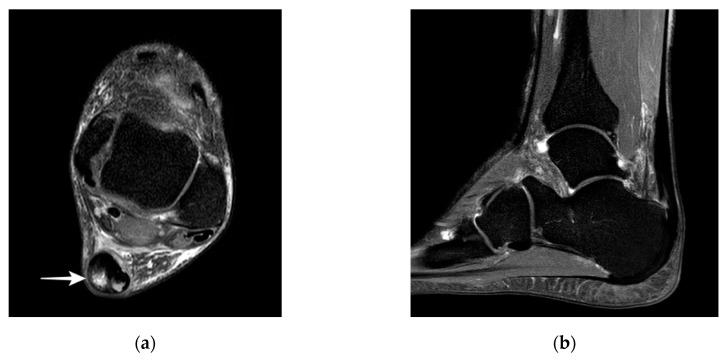
MR images of partial Achilles tendon ruptures: (**a**) partial rupture in the dorsal part of the midportion; (**b**) partial rupture in the insertion. MR; magnetic resonance.

The majority of studies report that with ultrasound it is difficult to distinguish partial ruptures from focal degenerative changes, since partial ruptures appear with a wavy, irregular echo pattern with accompanying focal hypoechogenic areas, detectable neovascularization, and tendon thickening that are also findings in Achilles tendinopathy [19,23,26,28,29]. A more specific finding might be a disrupted dorsal Achilles tendon border [19,28] (Figure 2a). The detection of proximal partial ruptures close to the myotendinous junction and the differentiation between older partial ruptures and intratendinous tendinopathy are challenging with ultrasound [28,30] (Figure 2b). In MRI, a partial tear is defined as tendon thickening with a hyperintense signal on T1 and a strong hyperintense signal on magnetic resonance (MR) images with fluid-sensitive (T2-weighted and inversion recovery) sequences [29,30,31] (Figure 3). Typically, signal intensity is analogous to free fluid. A hyperintense area directly located at the tendon border should be interpreted as a partial rupture [30]. Due to muscle inactivity, isolated fatty degeneration and edema in the calf muscles might represent a subsequent state of a partial or total rupture [31]. The sensitivity of MRI in detecting partial tears is high (positive predictive value 0.94, *n* = 18), but studies directly comparing the accuracy of MRI and ultrasound are rare [32]. In a prospective study of Kayser et al., only one-fifth of partial lesions were noticed with B-Mode ultrasound whereas MRI detected five-fifths of partial ruptures [24].

**Figure 4:**
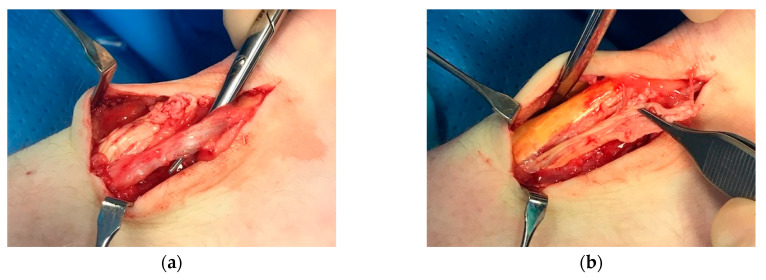
Patient with a partial rupture in close relation to a thick and compressing plantaris tendon: (**a**) thickened plantaris tendon compressing onto the medial side of the Achilles; (**b**) surgical release of the compressing plantaris tendon.

Another clinical observation from our patients is that the plantaris tendon may mimic pain from a partial Achilles tendon rupture. Typically, there is sudden sharp pain on the medial side of the Achilles, not seldom in the proximal or midportion Achilles tendon region (Figure 4). However, for plantaris-tendon-related pain the symptoms most often subside within a couple of days and then return during explosive plantar and dorsiflexion activities. Plantaris tendon involvement can be diagnosed using ultrasound [33,34,35].

### 3.6. Treatment Options

Based on the findings of the conducted systematic literature research, there is no clear consensus about a general conservative or surgical treatment regime. Indications for surgical treatment are functional limitations, such as poor single-heel raises and persistent pain in combination with positive findings in MRI or ultrasound [6,36]. In some studies, a partial tendon rupture of >50% of the cross-sectional area was seen as a criteria for surgical therapy, whereas minor rupture sizes, especially at the proximal myotendinous junction, with reduced biomechanical function might require surgical therapy as well [30,37].

#### 3.6.1. Nonsurgical Treatments

Studies on conservative treatments on patients with partial Achilles tendon tears are rare. The only case series found on exercise interventions was from Masci and colleagues [38]. All patients (*n* = 26) were clinically and through ultrasound examination diagnosed to have a partial Achilles tendon rupture. They underwent a 3-month structured rehabilitation program, starting with 6 weeks (phase 1) of heel lifts (2 cm) and avoiding calf stretching activities (for 12 weeks). Walking and moderate cycling with pain-adapted full weight-bearing were allowed. In phase II (weeks 7–12), heel lifts were reduced and concentric calf raises were introduced, starting with seated and progressing towards standing bilateral and single calf raises. After 3 months, heel lifts were removed and eccentric heel drops were introduced (Table 1). The clinical results were good for the majority of patients. In 25 of 26 patients, there was a significant pain reduction (Visual-Analogue Scale) and an improvement in the ultrasound and color Doppler findings after 3 months [37]. Recently, Medeiros presented in his case report of a futsal player with a partial rupture of up to 50% of the cross-sectional area a detailed rehabilitation protocol suggesting an initial immobilization with crutches and 2 cm heel lifts for 6 weeks combined with a progressive loading protocol [39].

Beyond physiotherapeutic approaches, extracorporeal shock wave therapy (ESWT) and platelet-rich plasma (PRP) injections are additional options. However, clinical results are based on single case reports and need to be evaluated in further studies [40,41]. Hence, for conservative treatment of partial ruptures there is no evidence-based approach available. The exercise protocol suggested by Masci and Alfredson is promising but needs to be evaluated in large-scale trials using specific patient-related outcome parameters [38].

#### 3.6.2. Surgical Treatments

In relation to surgical treatment, there are a few low-evidence studies in the current literature. In general, surgical treatment differs depending on the exact location of the partial rupture (insertion vs. midportion) within the tendon. In general, surgery can be divided into lesion excision with or without suture adaption and tendon augmentation or tendon transfer surgery depending on the size of the partial rupture. Using drill holes or anchors for reattachment for insertional ruptures is another option. There is no consensus for the optimal time for surgery, but in the majority of the studies operative treatment was initiated after a conservative approach. It is presumable that this might not affect patient outcomes, as seen in a recent study about a late minimally invasive Achilles tendon repair after 14 days [42]. Irrespective of the anatomic locations, there might be no relevant symptom improvement 3 months postoperatively, but there may be after 6–12 months with no deterioration after years [43].

With regard to partial ruptures in the midportion, previous studies combined heterogeneous patient cohorts suffering from total and partial ruptures with conservative and surgical therapy in their analyses, such that data interpretation is biased [20,44]. In a study of Denstad et al., 44 of 58 partial ruptures were treated with excision of pathologic tendon tissue and a suture side-to-side adaptation with a few (*n* = unknown) requiring a Lindholm repair with a turn down flap [20]. Overall, the results were good with 46 pleased, 8 satisfied, and 3 unsatisfied patients (1 lost to follow-up). There was a return-to-sports rate of (100%, *n* = 44) with 85% reaching an equal or better level [20]. Another study using the equal surgical procedure on 64 patients with a mean follow-up of 6 years reported a satisfaction rate of only 66% with a reoperation rate of 14% and more favorable results in proximal ruptures than distal or combined ruptures [45].

Regarding surgical treatment of insertional partial ruptures, prospective studies are rare. Jerosch et al. performed an endoscopic calcaneoplasty for insertional Achilles tendinopathy in 164 patients; 61 patients had a distal partial rupture based on MRI [46]. Based on the Ogilvie–Harris scores, 155 patients showed good or excellent results after 46 months. Even though the outcome of partial ruptures was not evaluated separately in this study, it indirectly indicates that suture application of partial ruptures might not be strictly necessary for achieving good long-term results [46].

Lohrer performed operations on 36 patients with persistent insertional Achilles pain with bursectomy and resection of Haglund exostosis; 5 out of 36 patients required a suture of a ventral longitudinal partial rupture based on the intraoperative surgeon’s decision [14]. However, there is no comparative data on whether or not sutures of this kind of tear are mandatory [14]. In a further study, anterior partial tears in 20 patients were resected and a tendon reconstruction was performed with sutures [47]. Postoperatively, patients were mobilized with a walking boot and decreasing heel support (2.5–1 cm) between the first and sixth week. The VISA-A score increased from 46 points to 72 after 6 months and to 84 points after 12 months [47].

An additional treatment strategy might be a bone–quadriceps tendon graft for insertional ruptures, which has been evaluated in a retrospective study on 24 patients with a high rate of satisfaction on the AOFAS-Score. There were few side effects—one wound healing problem and one instance of deep venous thrombosis [37]. Further treatment strategies might include augmentation with the plantaris tendon, flaps, or a flexor hallucis longus transfer [5,9].

## 4. Discussion and Clinical Recommendations

For partial Achilles tendon ruptures, there are few studies and unfortunately sparse knowledge of scientific value, making it difficult to give strict recommendations for diagnostics and treatment. High-quality research studies on this diagnosis are warranted.

Based on the presented studies and our clinical experience in the field of Achilles tendinopathy and partial Achilles tendon ruptures, diagnosis should be based on a patient’s history with a typical sharp onset of pain and inability to fully load the tendon. Clinical findings are a localized tender region in the tendon and often some weakness during heel raises. Greyscale ultrasound with a high-resolution probe and Power Doppler show a region with an irregular and bulging superficial tendon line. MRI shows a hyperintense signal on T1 and T2-weighted sequences. First-line therapy should be a conservative approach, such as using a 2 cm heel lift with pain-adapted full weight-bearing and avoiding tendon stretching for the first 6 weeks. This phase is followed by reduced heel lifts to 1 cm and progressive tendon loading in weeks 7–12. After 12 weeks, heel lifts can be removed if pain-free, and the patient can start eccentric exercises, lowering the heel under floor level, and gradually returning to the patient’s previous sport level. If conservative management has a poor effect, surgical exploration and excision of the partial rupture and suturing is required. However, this might only be needed in patients previously treated with intratendinous injections (most commonly cortisone). Augmentation procedures or anchor applications might be useful in some cases, but this depends on the size and location. After surgery, we recommend the 12 to 14-week rehabilitation program used in conservative management before a return to full tendon-loading activities.

Future research needs to provide basic epidemiological data about size, location, and affected patients. Additionally, imaging studies should focus on the diagnostic accuracy and monitoring capacities of MRI versus high-resolution ultrasound and further assessments of promising ultrasound technologies, such as shear wave elastography or ultrasound tissue characterization [48,49]. Evidence-based decision-making for conservative approaches requires prospective trials evaluating the effect of immobilization, heel raises, or physiotherapy [50]. Moreover, indications for surgical treatment, augmentation procedures, or suture application need to be studied in relation to the location and the size of the rupture.

## Figures and Tables

**Table 1:** Suggested rehabilitation program by Masci and Alfredson [38].

0–6 Weeks	7–12 Weeks	>12 Weeks (When Pain-Free)
• 2 cm heel lifts	• 1 cm heel lifts	• Heel lifts removed
• Avoiding calf muscle stretching	• Seated calf raises (3 × 15 reps daily) Progression to standing bilateral calf raises (from floor level to tiptoe, 3 × 15 reps daily) Progression to single calf raises (floor level to tip toe, 3 × 15 reps daily)	• Eccentric heel drops (from tiptoe to below floor level, 3 × 15 reps, 3 ×/week)
• Walking/cycling allowed	• Exercises performed 3 ×/week together with cross training, walking, swimming, and cycling	• Gradual return to previous (pre-injury) tendon loading
